# Mortality prediction in patients with hyperglycaemic crisis using explainable machine learning: a prospective, multicentre study based on tertiary hospitals

**DOI:** 10.1186/s13098-023-01020-1

**Published:** 2023-03-11

**Authors:** Puguang Xie, Cheng Yang, Gangyi Yang, Youzhao Jiang, Min He, Xiaoyan Jiang, Yan Chen, Liling Deng, Min Wang, David G. Armstrong, Yu Ma, Wuquan Deng

**Affiliations:** 1 Department of Endocrinology and Bioengineering College, Chongqing University Central Hospital, Chongqing Emergency Medical Centre, Chongqing University, NO. 1 Jiankang Road, Yuzhong District, Chongqing, 400014 China; 2 Department of Endocrinology, The Second Affiliated Hospital, Chongqing Medical University, Chongqing, 400010 China; 3 Department of Endocrinology, People’s Hospital of Chongqing Banan District, Chongqing, 401320 China; 4 General Practice Department, Chongqing Southwest Hospital, Chongqing, 400038 China; 5 Department of Surgery, Keck School of Medicine of University of Southern California, Los Angeles, CA 90033 USA

**Keywords:** Hyperglycaemic crisis, Mortality, Machine learning, Explainable model

## Abstract

**Background:**

Experiencing a hyperglycaemic crisis is associated with a short- and long-term increased risk of mortality. We aimed to develop an explainable machine learning model for predicting 3-year mortality and providing individualized risk factor assessment of patients with hyperglycaemic crisis after admission.

**Methods:**

Based on five representative machine learning algorithms, we trained prediction models on data from patients with hyperglycaemic crisis admitted to two tertiary hospitals between 2016 and 2020. The models were internally validated by tenfold cross-validation and externally validated using previously unseen data from two other tertiary hospitals. A SHapley Additive exPlanations algorithm was used to interpret the predictions of the best performing model, and the relative importance of the features in the model was compared with the traditional statistical test results.

**Results:**

A total of 337 patients with hyperglycaemic crisis were enrolled in the study, 3-year mortality was 13.6% (46 patients). 257 patients were used to train the models, and 80 patients were used for model validation. The Light Gradient Boosting Machine model performed best across testing cohorts (area under the ROC curve 0.89 [95% CI 0.77–0.97]). Advanced age, higher blood glucose and blood urea nitrogen were the three most important predictors for increased mortality.

**Conclusion:**

The developed explainable model can provide estimates of the mortality and visual contribution of the features to the prediction for an individual patient with hyperglycaemic crisis. Advanced age, metabolic disorders, and impaired renal and cardiac function were important factors that predicted non-survival.

*Trial Registration Number:* ChiCTR1800015981, 2018/05/04.

**Supplementary Information:**

The online version contains supplementary material available at 10.1186/s13098-023-01020-1.

## Introduction

Hyperglycaemic crisis is one of the most serious acute metabolic complications of diabetes and includes three subtypes: diabetic ketoacidosis (DKA), hyperosmolar hyperglycaemic state (HHS), and mixed syndrome (combined DKA-HHS) [1]. Inpatients with DKA or recurrent DKA are all at high risk for all-cause mortality [2]. Among diabetic patients, 10% of deaths are caused by confirmed or possible DKA or coma [3]. HHS is common in elderly patients with diabetes mellitus. Despite a relatively low incidence, the mortality of hospitalized patients with HHS can reach up to 10–20% [4, 5]. Alarmingly, the 30-day mortality in patients with combined features of DKA and HHS is approximately 2.7 times higher than that in patients with isolated hyperglycaemic crisis [6]. In addition to a high risk of short-term mortality, patients with hyperglycaemic crisis episode have a higher long-term mortality after hospital discharge [1, 7]. At present, due to the poor understanding of the pathogenesis of hyperglycaemic crisis and the complexity of treatment, there is a lack of powerful indicators for evaluating the risk of mortality in patients with hyperglycaemic crisis [6]. Predicting the risk of mortality as well as providing personalized analysis of the risk factors in patients with hyperglycaemic crisis at initial diagnosis may help physicians to make correct clinical judgments and select the most appropriate strategy of treatment.

Much effort has been put into the development of prediction models to predict the risk of mortality for patients with hyperglycaemic crisis. Traditionally, linear models, such as logistic regression model and Cox proportional hazard model, have been used to develop such prediction models [8–12]. Nevertheless, modernhigh-dimensional and incomplete medical data present a challenge to traditional statistical models, and the low precision of linear models impedes patient-level use. Lacking adequate prediction tools, physicians mainly rely on subjective judgement, which is prone to errors and biases. Previous studies have applied machine learning to establish models for predicting the clinical outcomes of patients with diabetic complications and achieved promising results [13–15]. Compared with linear models, machine learning models can provide more accurate prediction results by fitting high-dimensional and nonlinear relationships in the data [16–18]. However, most of the developed prediction models were opaque and unexplainable. The improvement of model performance also brings corresponding disadvantages: these models might be perceived as black-box models due to complex computational processes, meaning that the clinician can only see the input and output of the model, and it is difficult to understand how the predictions are generated, which could reduce their acceptance among clinicians [19]. In our recent study, we developed an explainable machine learning model for predicting amputation rate in patients with diabetic foot [20]. The proposal of algorithms that could provide explanations for black-box models might increase the understanding of the model predictions and facilitate clinicians in making more accurate decisions using machine learning models [21]. There is a lack of tools to predict long-term mortality in patients with hyperglycaemic crisis. In addition, to our knowledge, no study has developed a tool to use the machine learning model for predicting mortality in patients with hyperglycaemic crisis directly interpretable. Of note, the predictions of machine learning models are expected to be transparent to enable physicians to better understand and utilize these tools.

Here, we developed a machine learning model for predicting the 3-year mortality of inpatients with hyperglycaemic crises. In addition, we utilized a tool to interpret the developed black-box machine learning model to obtain a method for individualized mortality prediction and risk factor analysis for patients with hyperglycaemic crisis.

## Materials and methods

### Study design and participants

For model development and validation, we prospectively collected data from patients with hyperglycaemic crises who were hospitalized at four university-affiliated tertiary teaching hospitals between May 2016 and May 2020. All patients were followed up until May 2021. The 3-year mortality rate was chosen as the prediction target because the study was designed to predict the long-term mortality risk of patients, and most of the patients included in the study were followed up for 3 years. The study was conducted in accordance with the Declaration of Helsinki and protocols were approved by the Ethics Committee of Chongqing University Central Hospital. Inpatients aged 18 or older diagnosed with hyperglycaemic crisis were enrolled in the study. Case definition of hyperglycaemic crisis on admission was: (1) DKA: plasma glucose ≥ 13.9 mmol/L, positive urine ketones or serum ketones, and serum bicarbonate ion concentration (HCO3^−^) ≤ 18 mmol/L or hydrogen ion concentration index (PH) ≤ 7.3; (2) HHS: plasma glucose ≥ 33.3 mmol/L, small urine ketones or serum ketones, HCO3^−^  ≥ 15 mmol/L or PH ≥ 7.3, and effective serum osmolality ≥ 320 mOsm/kg; (3) combined DKA-HHS: plasma glucose ≥ 33.3 mmol/L, positive urine ketones or serum ketones, HCO3^−^  ≤ 18 mmol/L or PH ≤ 7.3, and effective serum osmolality ≥ 320 mOsm/kg. The effective serum osmolality was calculated from the following equation: 2 × [Na^+^ (mEq/L)] + [plasma glucose (mmol/L)].

The blood glucose, serum sodium, serum potassium, PH, HCO3−, base excess, serum creatinine, blood urea nitrogen, cystatin C, creatine kinase, total cholesterol, low-density lipoprotein cholesterol, triglyceride, C-reactive protein, procalcitonin, creatinine kinase MB isoenzyme (CK-MB), alanine aminotransferase, and aspartate aminotransferase were measured using an automatic biochemical analyzer (AU5821, Beckman Coulter, USA). Hemoglobin A1c was measured by high-performance liquid chromatography (BC-6800, Mindray, Shenzhen, China). Cardiac troponin I was measured by chemiluminescence immunoassay, using DXI800 (Beckman Coulter, USA).β-hydroxybutyrate was measured using colorimetric enzymatic reaction (D-3-hydroxybutyrate kit, Ranbut, Randox Laboratories). White blood cells, neutrophils, lymphocyte, and platelet were determined by an automatic blood cell analyzer (BC-6800, Mindray, Shenzhen, China).

There were 41 input variables incorporated into this study to determine the prediction models, including demographic features, clinical and at-admission laboratory data, and comorbidities. The data were collected by reviewing electronic medical records (EHRs). 9 patients with missing values of variables greater than 30% and 14 patients who were lost to follow-up were excluded. The participants were divided into two groups: data from two tertiary teaching hospitals were used as the training set for model training, and data from the two other tertiary teaching hospitals were used as the test set for external validation.

### Statistical analyses

Statistical analysis was applied among the groups. Continuous variables are presented as the mean ± standard deviation when data followed a normal distribution or as the median and interquartile range when the continuous variables were not found to be normal, and categorical variables were expressed as numbers (%). To evaluate significant differences between groups, the t test, Kruskal‒Wallis test, and chi-squared test were used for normal continuous, nonnormal continuous, and categorical variables, respectively. A *P* value < 0.05 was considered statistically significant.

### Model development

The data were preprocessed before training the prediction model. Box diagrams was used to eliminate outliers from the raw dataset, the k-nearest neighbour algorithm was used for filling in the missing values, and the Z Score was utilized to normalize continuous variables.. To construct the prediction model, five algorithms were used: logical regression (LR), support vector machine (SVM), random forest (RF), light gradient boosting machine (LightGBM), and deep neural networks (DNNs). The DNN architecture used in this study was a feed-forward neural network. LR and SVM are classical machine learning algorithms commonly used in previous studies [22, 23]. RF and LightGBM are powerful algorithms in the machine learning area and are currently considered state-of-the-art algorithms for prediction with tabular data [24–26]. DNNs belong to an important branch of machine learning algorithms, achieving excellent performance in many fields, such as pattern recognition and natural language processing [18, 27]. Therefore, the above five algorithms were used for model building. Afterwards, tenfold cross-validation and Bayesian hyperparameter optimization were utilized for model training, internal validation and hyperparameter tuning. The generalization capacity of hyperparameter combinations was improved by tenfold cross-validation, and the efficiency of finding the optimal hyperparameter combination was improved by Bayesian hyperparameter optimization. Additional details on hyperparameter setting and model architecture are provided in Additional file 1: Fig. S1 and Tables S1–S6.

After model training was performed, the prediction ability of the models was evaluated and compared in the test set according to the evaluation metrics, including area under the receiver-operating-characteristic curve (AUC), sensitivity, specificity, positive predictive value (PPV) and negative predictive value (NPV). Confidence intervals (CIs) were obtained by resampling the test set 1000 times (bootstrapping) and averaging the performance.

The best performing model was selected as the prediction model, and Platt scaling was further applied to calibrate the predicted probability of the best performing model to make it close to the observed probabilities. The test set was further divided in a 1:1 ratio into a calibration set for model calibration and a new test set for evaluating the performance of model calibration. The calibration curve and Brier score were used to assess the coherence between the predicted and observed probabilities of the prediction model.

### Model explanation

The Shapley additive explanations (SHAP) algorithm was applied to the calibrated model to obtain explanations of the predictions of the model. The SHAP algorithm is one of the most popular model-agnostic algorithms for interpreting black-box model predictions [28, 29]. SHAP values were obtained by the SHAP algorithm, which provides interpretation of individual predictions. A SHAP value represents, given a set of feature values, how much a single feature value influences the difference between the actual prediction and the average prediction in its interaction with other feature values. Therefore, the mean prediction of the model plus the sum of the SHAP values for all features are consistent with the predicted result. Importantly, the SHAP value for a feature is not isolated but obtained by interacting with other features, which makes it different from the feature weight in the traditional generalized linear model.

To verify the rationality of the interpretation of the model predictions acquired by the SHAP algorithm, we first utilized the SHAP algorithm to obtain and visualize the overall effect of features on the predictions, that is, the contribution and relative importance ranking of each feature to the predictions. For further validation and comparison, we divided the patients in the training set into a survival group and a nonsurvival group according to their clinical outcomes and analysed the differences in features between the groups by statistical methods. In addition, based on proving the rationality of the explanations obtained by the SHAP algorithm, we further linearly mapped SHAP values to the probability of increased or decreased mortality and proposed a personalized mortality risk factor analysis method specific to patients with hyperglycaemic crisis, which visualized the contribution of each feature to the prediction in probability.

## Results

**Fig. 1:**
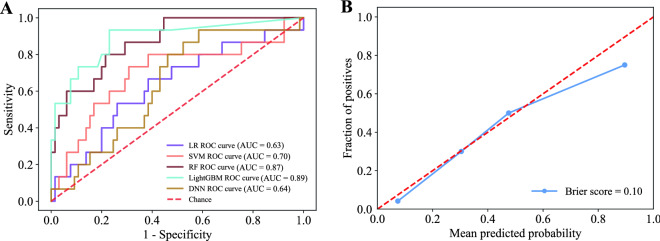
Discrimination and calibration performance of the models. **A** Receiver operating characteristic curves for the LR, SVM, RF, LightGBM, and DNN models. **B** Calibration curve for the LightGBM model

For model development, 257 patients with hyperglycaemic crisis from two hospitals were enrolled. The baseline characteristics of these patients are depicted in Table 1. In the training set, the median age was 56 years (IQR 40.3–70.0), and 152 (59.1%) were male. Death occurred in 31 (12.1%) patients within the study period. To evaluate the external validity, the models were applied in the external test set, comprising 80 patients with hyperglycaemic crisis from two hospitals that were independent of the training set. In the test set, the receiver operating characteristic curve and AUC of the five models are shown in Fig. 1A (AUC = 1 indicates perfect prediction; AUC = 0 indicates random prediction). The other five evaluation metrics, including accuracy, sensitivity, specificity, NPV and PPV, for the five models are presented in Table 2. Overall, the findings demonstrated that the LightGBM model performed best among the five prediction models, with an AUC of 0.89 (95% CI 0.77, 0.97). The corresponding accuracy was 0.83 (0.74, 0.90), sensitivity was 0.74 (0.47, 0.94), specificity was 0.85 (0.76, 0.93), PPV was 0.52 (0.31, 0.74), and NPV was 0.94 (0.87, 0. 99). Therefore, the LightGBM model was selected as the best predictive model. The prediction probability of the LightGBM model was calibrated to make it close to the observed probability. The calibration plot indicated good agreement between the predicted and observed probabilities of the LightGBM model with a curve close to the 45° line, and the Brier score was 0.10 (0.05, 0.17) (Fig. 1B).

**Table 1:** Baseline characteristics of patients with hyperglycaemic crisis in the training set and test set

Variables	Training set (n = 257)	Test set (n = 80)
*Demographic data*		
Age, years	56.0 (40.3, 70.0)	51.0 (35.0, 61.0)
Sex, %		
Male	152 (59.1)	46 (57.5)
Female	105 (40.9)	34 (42.5)
Body mass index, kg/m^2^	22.7 (20.1, 25.0)	22.8 (20.6, 26.1)
Diabetes type, %		
Type 1	37 (14.4)	14 (17.5)
Type 2	220 (85.6)	66 (82.5)
*Clinical and laboratory data*		
Blood glucose, mmol/L	33.1 (21.6, 33.6)	27.3 (20.6, 40.2)
β-hydroxybutyrate, mmol/L	3.80 (1.50, 6.00)	5.60 (3.40, 6.85)
Hemoglobin A1c, %	11.6 (10.0, 13.5)	12.8 (11.0, 14.3)
Triglyceride, mmol/L	1.95 (1.33, 3.02)	1.62 (1.10, 3.30)
Total cholesterol, mmol/L	4.80 (3.80, 6.27)	4.50 (3.53, 5.51)
LDL-C, mmol/L	2.57 (1.78, 3.37)	2.53 (1.66, 3.19)
Serum creatinine, umol/L	82.0 (58.0, 144.1)	79.5 (57.6, 120.8)
Blood urea nitrogen, mmol/L	7.86 (5.40, 15.40)	7.81 (5.15, 16.10)
Cystatin C, mg/L	1.16 (0.71, 2.20)	0.74 (0.61, 1.21)
Creatine kinase, U/L	95.0 (58.0, 208.0)	83.0 (46.6, 123.6)
Cardiac troponin I, μg/L	0.01 (0.00, 0.04)	0.01 (0.01, 0.03)
CKMB, U/L	14.0 (8.3, 20.8)	16.6 (11.2, 25.6)
Alanine aminotransferase, IU/L	19.0 (13.0, 30.0)	18.0 (12.1, 29.6)
Aspartate aminotransferase, IU/L	18.0 (13.0, 28.3)	17.8 (13.2, 26.8)
C-reactive protein, mmol/L	9.10 (4.20, 55.66)	4.56 (0.66, 14.30)
Procalcitonin, ng/ml	0.47 (0.08, 2.25)	0.15 (0.10, 2.20)
White blood cells, × 10^9^/L	10.6 (6.8, 14.8)	7.24 (10.89, 15.71)
Percentage of neutrophils, %	84.0 (74.0, 90.0)	86.5 (78.6, 91.4)
Lymphocyte, × 10^9^/L	1.06 (0.62, 1.59)	1.23 (0.76, 1.91)
Platelet, × 10^9^/L	196.0 (153.0, 259.0)	214.5 (169.8, 284.3)
Serum sodium, mmol/L	142.3 (135.2, 149.5)	136.5 (133.0, 142.0)
Serum potassium, mmol/L	4.03 (3.66, 4.66)	4.15 (3.64, 4.92)
Serum chloride, mmol/L	100.1 (95.8, 106.0)	104.5 (97.8, 112.0)
PH	7.31 (7.22, 7.38)	7.30 (7.19, 7.36)
Base excess, mmol/L	− 7.30 (− 15.70, − 2.70)	− 9.90 (− 17.83, − 6.55)
HCO3^−^, mmol/L	14.9 (9.2, 18.0)	14.5 (8.3, 16.9)
Effective serum osmolality, mOsm/kg	314.0 (293.0, 335.5)	299.0 (289.0, 327.2)
*Medical history*		
Infection, n (%)	147 (57.2)	30 (37.5)
Septic shock, n (%)	7 (2.7)	0 (0.0)
Hypertension, n (%)	66 (25.7)	16 (20.0)
Coronary heart disease, n (%)	29 (11.3)	8 (10.0)
Heart failure, n (%)	12 (4.7)	0 (0.0)
Cerebral infarction, n (%)	44 (17.1)	4 (5.0)
Dementia, n (%)	5 (1.9)	0 (0.0)
Diabetic nephropathy, n (%)	43 (16.7)	11 (13.8)
Acute kidney injury, n (%)	8 (3.1)	6 (7.5)
Tumor, n (%)	3 (1.2)	3 (3.8)
Death, n (%)	31 (12.1)	15 (18.8)

*LDL-C* low-density lipoprotein cholesterol, *CKMB* creatinine kinases MB isoenzyme, *PH* hydrogen ion concentration index, *HCO3*^*−*^ serum bicarbonate ion concentration

**Table 2:** The values of the evaluation metrics of the models in the test set

	AUC	Accuracy	Sensitivity	Specificity	PPV	NPV
LR	0.64 (0.47, 0.79)	0.76 (0.69, 0.85)	0.20 (0.00, 0.44)	0.89 (0.81, 0.96)	0.29 (0.00, 0.60)	0.83 (0.74, 0.92)
SVM	0.70 (0.52, 0.86)	0.76 (0.65, 0.85)	0.47 (0.22, 0.73)	0.83 (0.73, 0.91)	0.39 (0.18, 0.63)	0.87 (0.77, 0.95)
RF	0.87 (0.78, 0.95)	0.80 (0.70, 0.88)	0.67 (0.42, 0.91)	0.83 (0.73, 0.91)	0.48 (0.28, 0.69)	0.92 (0.85, 0.98)
LightGBM	**0.89 (0.77, 0.97)**	**0.83 (0.74, 0.90)**	**0.74 (0.47, 0.94)**	0.85 (0.76, 0.93)	**0.52 (0.31, 0.74)**	**0.94 (0.87, 0. 99)**
DNN	0.64 (0.54, 0.87)	0.81 (0.73, 0.89)	0.26 (0.06, 0.53)	**0.94 (0.88, 0.99)**	0.50 (0.11, 0.88)	0.85 (0.76, 0.92)

*LR* logical regression, *SVM* support vector machine, *RF* random forest, *LightGBM* light gradient boosting machine, *DNN* deep neural network algorithm, *NPV* negative predictive value, *PPV* positive predictive value

**Fig. 2:**
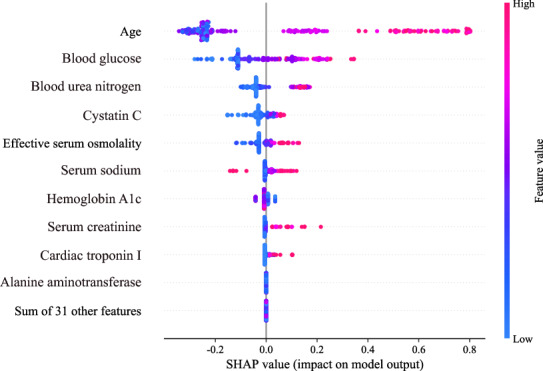
The impact of the input features on predictions. Each dot represents the effect of a feature on the prediction for one patient. The redder the colour of the dots, the higher the value of the features, and the bluer the colour of the dots, the lower the value of the features. Dots to the left x-axis represent patients with values of the features decreasing mortality prediction, and dots to the right x-axis represent patients with values of the features increasing mortality prediction

**Fig. 3:**
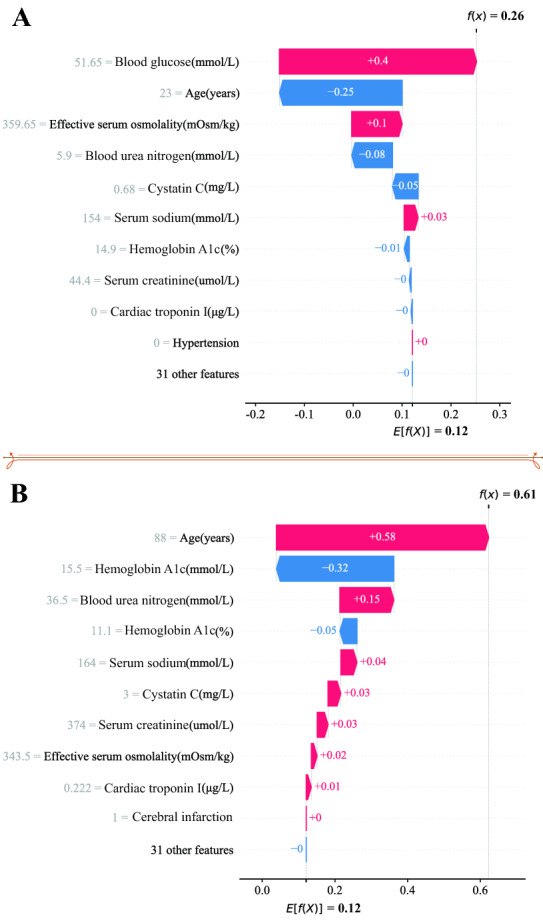
Examples of personalized risk factors. **A** An example of personalized risk factor analysis for a patient in the test set (clinical outcome was death). **B** An example of personalized risk factor analysis for a patient in the test set (actual clinical outcome was survival)

The contribution of each of the 41 features in the calibrated LightGBM model is shown in Fig. 2. The features were ranked by their relative importance to mortality prediction according to the SHAP values of the model predictions. It is not surprising that age was ranked as the most important feature for the prediction model, followed by blood glucose and blood urea nitrogen. In addition, taking the effect of age on the prediction as an example, older age was associated with a higher risk of death, and younger age drives the predictions towards survival. A similar explanation can be applied to other features, and most of the interpretation of features was consistent with clinical experience and previous evidence. Of note, features can drive the prediction in either direction (increase or decrease mortality prediction) in our explainable prediction model, which is different from the previous mortality risk scoring system based on a generalized linear model in which features can only drive mortality prediction in a single direction. As shown in Table 3, the results of statistical analysis revealed that the 9 most important features for the LightGBM model were significantly different between the survival group and the nonsurvival group in the training set (P < 0.05). Compared to the survival group, age, blood glucose, serum creatinine, blood urea nitrogen, cystatin C, effective serum osmolality, CK-MB, alanine aminotransferase, serum sodium, PH, HCO3 − and cardiac troponin I were significantly higher in the nonsurvival group (P < 0.05). However, hemoglobin A1c level was surprisingly significantly lower in the nonsurvival group than survival group (P < 0.05). An increasing trend of β-hydroxybutyrate was unexpectedly indicated in the survival group (P = 0.045). Therefore, the traditional statistical test results and the model interpretation results corroborated each other, which proved the rationality and accuracy of the interpretation of features acquired by the SHAP algorithm. Based on this evidence, we mapped SHAP values and proposed a personalized risk factor analysis tool for explaining the mortality prediction for a particular patient with hyperglycaemic crisis, which is a scale from 0 to 1, visualizing the contribution of each feature to the prediction in probability. We showed the application of the personalized risk factor analysis method in one deceased and one surviving patient with hyperglycaemic crisis during the follow-up period in the test set (Fig. 3). In the case of the deceased patient, the patient was an 88-year-old female with a history of septic shock and acute kidney injury. The model predicted that the risk of mortality of the patients was 0.623. Advanced age (88 years) drove a 0.58 increase in the risk of mortality, while relatively low hemoglobin A1c reduced the risk of mortality by 0.32. A similar explanation can be applied to other features. The prediction was driven by 41 features used for model training. The sum of the SHAP values for all features plus the baseline risk equals the predicted risk of mortality. The baseline risk (E[f(X)]) was obtained by calculating the average predicted risk of mortality among all patients in the training set. Thus, SHAP algorithm made our model explainable both in terms of the relative importance of individual features for survival of patient with hyperglycaemic crisis and those at patient level.

**Table 3:** Baseline characteristics of patients with hyperglycaemic crisis in the training set by clinical outcomes

Variables	Survival group (n = 226)	Nonsurvival group (n = 31)	
*Demographic data*			
Age, years	54.5 (37.0, 67.3)	79.5 (65.0, 86.3)	** < 0.001**
Sex, %			0.194
Male	137 (60.6)	15 (48.4)	
Female	89 (39.4)	16 (51.6)	
Body mass index, kg/m^2^	22.8 (20.2, 25.0)	17.5 (20.7, 25.0)	0.076
Diabetes type, %			**0.015**
Type 1	37 (16.4)	0 (0.0)	
Type 2	189 (83.6)	31 (100.0)	
*Clinical and laboratory data*			
Blood glucose, mmol/L	30.1 (20.2, 33.3)	33.3 (33.3, 38.4)	** < 0.001**
β-hydroxybutyrate, mmol/L	4.13 (1.51, 6.20)	3.05 (0.48, 4.60)	**0.045**
Hemoglobin A1c, %	11.8 (10.2, 13.6)	10.6 (8.6, 12.5)	**0.038**
Triglyceride, mmol/L	1.89 (1.31, 3.11)	2.10 (1.40, 2.71)	0.386
Total cholesterol, mmol/L	4.80 (3.78, 6.26)	4.39 (3.80, 6.30)	0.675
LDL-C, mmol/L	2.52 (1.73, 3.35)	2.86 (2.01, 3.74)	0.159
Serum creatinine, umol/L	75.5 (56.8, 131.3)	165.0 (87.2, 334.8)	** < 0.001**
Blood urea nitrogen, mmol/L	7.50 (5.18, 13.31)	19.5 (11.0, 28.2)	** < 0.001**
Cystatin C, mg/L	1.10 (0.69, 1.88)	1.88 (1.28, 3.64)	** < 0.001**
Creatine kinase, U/L	50.0 (25.0, 85.1)	32.8 (5.8, 139.8)	0.306
Cardiac troponin I, μg/L	58.5 (30.3, 109.0)	44.0 (20.5, 177.8)	0.477
CKMB, U/L	87.0 (55.0, 187.0)	150.0 (76.5, 571.3)	**0.008**
Alanine aminotransferase, IU/L	0.01 (0.00, 0.03)	0.04 (0.00, 0.10)	**0.021**
Aspartate aminotransferase, IU/L	18.0 (12.0, 28.0)	19.0 (14.0, 31.0)	0.200
C-reactive protein, mmol/L	7.90 (3.81, 47.45)	19.1 (4.8, 145.0)	**0.048**
Procalcitonin, ng/ml	0.40 (0.05, 2.14)	0.71 (0.11, 6.80)	0.063
White blood cells, × 10^9^/L	10.5 (6.7, 14.6)	12.7 (7.1, 17.8)	0.271
Percentage of neutrophils, %	83.7 (73.9, 89.7)	84.1 (76.7, 93.4)	0.136
Lymphocyte, × 10^9^/L	1.08 (0.63, 1.60)	1.00 (0.54, 1.50)	0.513
Platelet, × 10^9^/L	202.0 (153.0, 261.5)	179.0 (133.5, 207.3)	0.085
Serum sodium, mmol/L	140.3 (134.7, 148.2)	149.0 (142.5, 159.4)	**0.001**
Serum potassium, mmol/L	4.03 (3.68, 4.66)	4.02 (3.62, 4.75)	0.759
Serum chloride, mmol/L	99.9 (95.6, 105.5)	102.5 (96.8, 111.7)	0.220
PH	7.30 (7.22, 7.37)	7.38 (7.25, 7.41)	**0.030**
Base excess, mmol/L	− 7.50 (− 2.70, − 16.43)	− 7.00 (− 2.50, − 12.00)	0.682
HCO3^−^, mmol/L	14.6 (7.8, 18.0)	17.6 (13.8, 21.2)	**0.031**
Effective serum osmolality, mOsm/kg	310.2 (292.0, 330.3)	327.0 (322.0, 353.0)	** < 0.001**
*Medical history*			
Infection, n (%)	122 (54.0)	25 (80.6)	**0.005**
Septic shock, n (%)	5 (2.2)	2 (6.5)	0.174
Hypertension, n (%)	57 (25.2)	9 (29.0)	0.649
Coronary heart disease, n (%)	24 (10.6)	5 (16.1)	0.363
Heart failure, n (%)	10 (4.4)	2 (6.5)	0.616
Cerebral infarction, n (%)	32 (14.2)	12 (38.7)	**0.001**
Dementia, n (%)	3 (1.3)	2 (6.5)	0.053
Diabetic nephropathy, n (%)	34 (15.0)	9 (29.0)	0.050
Acute kidney injury, n (%)	6 (2.7)	2 (6.5)	0.254
Tumor, n (%)	3 (1.3)	0 (0.0)	0.519

*LDL-C* low-density lipoprotein cholesterol, *CKMB* creatinine kinases MB isoenzyme, *PH* hydrogen ion concentration index, *HCO3*^*−*^ serum bicarbonate ion concentration. *P* value < 0.05 was considered statistically significant

## Discussion

Experiencing a hyperglycaemic crisis is associated with a short- and long-term increased risk of mortality [1, 6, 7]. However, due to the complex pathogenesis of hyperglycaemic crisis, available international guidelines for the diagnosis and treatment of hyperglycaemic crisis are not consistent [4, 30]. In addition, there is a lack of strong indicators to assess the risk of mortality in patients with hyperglycaemic crisis. Therefore, the development of more effective methods to predict the risk of mortality, create individualized risk and benefit evaluations in patients with hyperglycaemic crises at initial diagnosis, which are particularly important to identify the best therapeutic strategies and improve the prognosis.

Here, we developed an explainable risk prediction model providing predictions and individualized risk factor assessment of the 3-year mortality of patients with hyperglycaemic crisis after admission. In the model building process, we selected five representative machine learning algorithms, including LR, SVM, RF, LightGBM, and DNN, to obtain the best prediction model. The LightGBM model performed the best of the five models evaluated in an external test set, with an AUC of 0.89. We further calibrated the LightGBM model to obtain a more reliable model. The SHAP algorithm was used to interpret the calibrated LightGBM model to obtain how each feature drives the prediction of the model. On the basis of verifying the effectiveness of the analytical method by comparing with the statistical test results, we further proposed a personalized mortality risk factor assessment method specific to patients with hyperglycaemic crisis. In the interpretation obtained by SHAP algorithm, the influence of each feature on the predictions is not isolated, but interacts with other features, which is related to the calculation method of SHAP value, and makes it different from the feature weight in the traditional generalized linear model.Thus, the developed explainable model can not only predict mortality but also provide a personalized risk factor assessment tool. Such an explainable model is a more useful tool than scoring systems based on generalized linear models that are currently implemented.

Most of the prediction tools constructed in past studies are based on generalized linear models, such as logistic regression models and Cox proportional hazard models [10, 11, 31]. However, the rapid development of information technology brings high-dimensional and nonlinear data, which challenges the traditional generalized linear model. Machine learning provides a powerful and novel method to extract information from complex medical data and develop more accurate predictions. That is, we can only obtain the input of the model and the output of the predictions. It is difficult to understand the details of how machine learning models analyse data and make decisions, which limited the application of the models at the individual level. A representative score called PHD was developed based on a generalized linear model by Huang et al. [10], which could be used to predict 30-day mortality risk and classify risk and disposition in patients with hyperglycaemic crisis. Since the variables we selected were different from the PHD score, the model we developed predicted the 3-year mortality of patients with hyperglycaemic crisis after admission. Therefore, we could not directly compare our model with the PHD score. However, an external validation study revealed that the AUC of the PHD score ranged from 0.357 to 0.727 [9]. In comparison, the AUC of the models developed in this study ranged from 0.63 to 0.89 in an external validation dataset. In addition, the developed LightGBM model also outperformed the conventional logistic regression model constructed in our study in the external test data (Fig. 1A, AUC of 0.89 vs. 0.63). We thus consider our model superior to traditional methods.

In addition, we used the SHAP algorithm to explain the black-box model to quantify and visualize the features that drive the predictions so that it not only had better prediction ability but also had transparency similar to that of the simple linear model. The tools established in this study combined the advantages of the complex machine learning model and simple linear model, solving the problems of insufficient prediction ability of the generalized linear model and black box nature of the machine learning model. The model we developed provided explanations of the risk factors that drive the model prediction, both in terms of the importance of individual features to the overall mortality prediction and contribution at the patient level. A comprehensible model allows clinicians to combine the predictions with their expertise to facilitate decision-making and assist clinicians in interventions [32, 33].

The effect of most features on the prediction is consistent with clinical experience and previous evidence. For example, advanced age, metabolic disorders, and impaired renal and cardiac function can predict for nonsurvival. Advanced age was the most important risk factor for mortality. There is substantial evidence that the physical function and resistance of patients decrease with age, which is more likely to increase the risk of mortality [12, 34, 35]. Severe metabolic disorders (elevated levels of blood glucose, effective serum osmolality, and serum sodium) may lead to confusion and even coma, which is associated with an increased risk of mortality [5, 36]. Likewise, there is consistent evidence that impaired renal (elevated levels of blood urea nitrogen, cystatin C, and serum creatinine,) and cardiac function (elevated levels of cardiac troponin I) increase the risk of mortality [1, 34, 37–39]. In addition, reduced levels of HbA1c drove the prediction towards nonsurvival. The effect of HbA1c on the prediction did not seem to live up to expectations. One reason for this counterintuitive issue might be that patients in the survival group had significantly better renal function than those in the nonsurvival group, and there is evidence that patients with chronic renal failure generally had a lower red blood cell (RBC) survival rate [40]. In addition, after treatment with erythropoietin, the newly generated RBCs lead to a further decrease in HbA1c [41]. An increasing trend of β-hydroxybutyrate was unexpectedly found in the survival group. It seems that it is a protective factor for patients with hyperglycaemic crisis. Previous studies evidence supports that blood β-hydroxybutyrate can reduce renal ischemia and reperfusion injury by increasing the upstream regulator forkhead transcription factor O3 and reducing caspase-1 and pro-inflammatory cytokines, thereby reducing cell death [42, 43].

Age was ranked as the most important feature for the model, followed by features related to metabolic disorders, cardiac and renal dysfunction. In a recent study, acute hyperglycaemic crisis episode impact on survival in individuals with diabetic foot ulcer using a machine learning approach, which also revealed that individual characteristics evaluated by Charlson Comorbidity Index (CCI) and acute organ injury played a vital role in disease prognosis [44]. The nine most important features for the prediction were significantly different between the survival group and nonsurvival group in the training set. Therefore, the effect of features on the predictions is consistent with the traditional statistical test results. Importantly, the developed explainable model can provide the relative importance of individual features for survival of patient and those at patient level, which makes it superior to traditional statistical tests that can only test for significant differences between groups. Admittedly, there are some limitations in our study. First, although multicentre data were used, due to the low morbidity of hyperglycaemic crisis, the amount of data was relatively small, which may lead to bias in the model. Second, in order to enable the models to obtain more comprehensive information and improve the performance of the tree-based models, our models contained up to 41 features. However, due to the limitation of data acquisition, the number of variables selected for the study is limited.. Third, the SHAP algorithm cannot address model bias, and the influence of features on the predictions is not equal to the association in the causal chain. Finally, Although the model is explainable, some features, such as age, cannot be manipulated by physicians. However, these insights into the relationship between features and predictions may guide our search for causality.

## Conclusions

In summary, we developed an explainable machine learning model for predicting 3-year mortality and providing individualized risk factor assessment of inpatients with hyperglycaemic crises as well as hospital discharge, and the model was externally validated in an independent dataset. The interpretation results of the model revealed that more attention should be given to the variables related to metabolism and renal and cardiac function in the treatment of hyperglycaemic crisis, which played an important role in mortality through the model prediction. Transparent and explainable model predictions would help gain the trust of clinicians and facilitate decision-making by allowing physicians to evaluate whether the decision-making process of the model is consistent with scientific evidence and clinical experience. However, before this kind of tool used in the clinic, prospective studies are needed to be verified in the future.

## Supplementary Information

**Additional file 1: Table S1.** The hyperparameter optimization range and results of LR. **Table S2.** The hyperparameter optimization range and results of SVM. **Table S3.** The hyperparameter optimization range and results of RF. **Table S4.** The hyperparameter optimization range and results of LightGBM. **Figure S1.** Schematic diagram of DNN network structure. **Table S5.** The hyperparameter of the network structure of DNN. **Table S6.** The hyperparameter optimization range and results of DNN.

## Data Availability

All datasets generated during and analyzed during the current study are not publicly available but are available from the corresponding author on reasonable request.
