# Supplementary material for: Mortality prediction in patients with hyperglycaemic crisis using explainable machine learning: a prospective, multicentre study based on tertiary hospitals
Source: Diabetol Metab Syndr. 2023 Mar 11;15:44. doi: 10.1186/s13098-023-01020-1 (PMC10007769; doi:10.1186/s13098-023-01020-1)
Supplement: Supplementary file 1 — Additional file 1: Table S1. The hyperparameter optimization range and results of LR. Table S2. The hyperparameter optimization range and results of SVM. Table S3. The hyperparameter optimization range and results of RF. Table S4. The hyperparameter optimization range and results of LightGBM. Figure S1. Schematic diagram of DNN network structure. Table S5. The hyperparameter of the network structure of DNN. Table S6. The hyperparameter optimization range and results of DNN. [file 13098_2023_1020_MOESM1_ESM.docx]

**Additional material**

**Mortality Prediction in Patients with** **Hyperglycaemic Crisis Using Explainable Machine Learning: A Prospective, Multicentre Study Based on Tertiary Hospitals**

*Puguang Xie^1^,* *Cheng Yang^1^,* *Gangyi Yang^2^,* *Youzhao Jiang ^3^,* *Min He ^4^,* *Xiaoyan Jiang^1^,* *Yan Chen^1^,* *Liling Deng^1^,* *David G. Armstrong^5^, Yu Ma^1^, Wuquan Deng^1^*

1. Department of Endocrinology & Bioengineering College, Chongqing University Central Hospital, Chongqing University, Chongqing 400014, China.

2. Department of Endocrinology, The Second Affiliated Hospital, Chongqing Medical University, Chongqing 400010, China.

3. Department of Endocrinology, People's Hospital of Chongqing Banan District, Chongqing 401320, China.

4. General Practice Department, Chongqing Southwest Hospital, Chongqing 400038, China.

5. Department of Surgery, Keck School of Medicine of University of Southern California, Los Angeles, CA 90033, USA.

**Corresponding authors:**

Wuquan Deng, Department of Endocrinology, Chongqing University Central Hospital

No.1 Jiankang Road, Yuzhong District, Chongqing 400014, China, phone: +86 23 63692185, Email: wuquandeng@cqu.edu.cn;

Yu Ma, Department of Endocrinology, Chongqing University Central Hospital

No.1 Jiankang Road, Yuzhong District, Chongqing 400014, China, phone: +86 23

63692002, Email: [81846846@qq.com](mailto:81846846@qq.com).

**Table S1: The hyperparameter optimization range and results of LR.**

| Hyper-parameter | Tuning range | Tuning result |
| --- | --- | --- |
| *c* | (0.001, 1000) | 55.96 |
| penalty | / | *l*_2_ |
| solver | / | liblinear |

LR, logical regression.

**Table S2:** **The hyperparameter optimization range and results of SVM.**

| Hyper-parameter | Tuning range | Tuning result |
| --- | --- | --- |
| *c* | (0.001, 1000) | 0.12 |
| gamma | (0.001, 0.1) | 0.03 |
| kernel | / | RBF |

SVM, support vector machine.

**Table S3: The hyperparameter optimization range and results of RF**

| Hyper-parameter | Tuning range | Tuning result |
| --- | --- | --- |
| n_estimators | (50, 500) | 106 |
| max_depth | (1, 10) | 7 |
| max_features | (5, 15) | 6 |
| min_samples_split | (2, 10) | 8 |
| min_samples_leaf | (1, 5) | 3 |

RF, random forest.

**Table S4:** **The hyperparameter optimization range and results of LightGBM.**

| Hyper-parameter | Tuning range | Tuning result |
| --- | --- | --- |
| learning_rate | (0.001, 1) | 0.09 |
| n_estimators | (50, 500) | 461 |
| subsample | (0.8, 1,0) | 0.79 |
| colsample_bytree | (0.8, 1,0) | 0.65 |
| reg_alpha | (0.01, 100) | 7.63 |
| reg_lamba | (0.01, 100) | 1.12 |
| max_depth | (1, 10) | 5 |
| num_leaves | (5, 15) | 16 |
| min_child_samples | (5, 30) | 4 |

LightGBM, light gradient boosting machine.


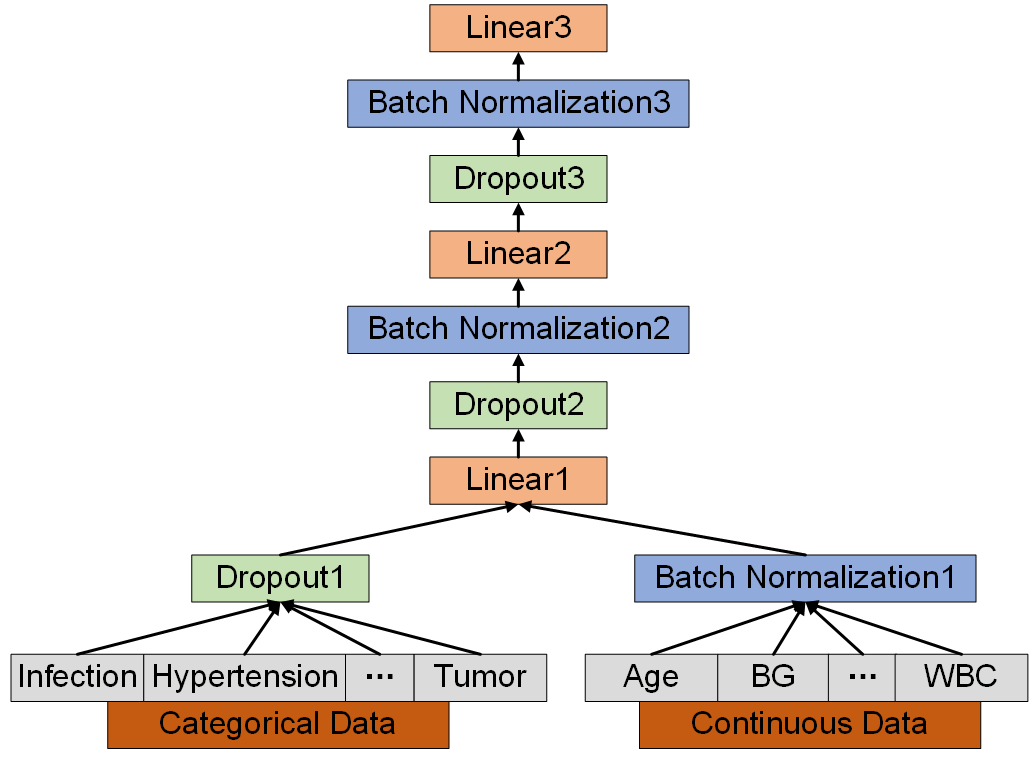


**Figure S1: Schematic diagram of DNN network structure**

**Table S5: The hyperparameter of the network structure of DNN.**

| Parameter | Value |
| --- | --- |
| Categorical/Dropout1 | 12 |
| Continuous/ Batch Normalization1 | 29 |
| Linear1/Dropout2/ Batch Normalization2 | 200 |
| Linear2/Dropout3/ Batch Normalization3 | 50 |
| Linear3 | 2 |
| Dropout1/ Dropout2/ Dropout3 | 0.5 |
| Activation function type | Relu |

DNN, deep neural network algorithm.

**Table S6:** **The hyperparameter optimization range and results of DNN.**

| Hyper-parameter | Tuning range | Tuning result |
| --- | --- | --- |
| learning_rate | (0.001, 0.1) | 0.08 |
| momentum | (0.8, 1) | 0.92 |
| optimzier | / | SGD |

DNN, deep neural network algorithm.
